# Identification of Novel Human Dipeptidyl Peptidase-IV Inhibitors of Natural Origin (Part I): Virtual Screening and Activity Assays

**DOI:** 10.1371/journal.pone.0044971

**Published:** 2012-09-12

**Authors:** Laura Guasch, Maria José Ojeda, Noemí González-Abuín, Esther Sala, Adrià Cereto-Massagué, Miquel Mulero, Cristina Valls, Montserrat Pinent, Anna Ardévol, Santiago Garcia-Vallvé, Gerard Pujadas

**Affiliations:** 1 Grup de Recerca en Nutrigenòmica, Departament de Bioquímica i Biotecnologia, Universitat Rovira i Virgili, Campus de Sescelades, Tarragona, Catalonia, Spain; 2 Centre Tecnològic de Nutrició i Salut, TECNIO, Campus of International excellence southern catalonia, Avinguda Universitat, Catalonia, Spain; University of South Florida College of Medicine, United States of America

## Abstract

**Background:**

There has been great interest in determining whether natural products show biological activity toward protein targets of pharmacological relevance. One target of particular interest is DPP-IV whose most important substrates are incretins that, among other beneficial effects, stimulates insulin biosynthesis and secretion. Incretins have very short half-lives because of their rapid degradation by DPP-IV and, therefore, inhibiting this enzyme improves glucose homeostasis. As a result, DPP-IV inhibitors are of considerable interest to the pharmaceutical industry. The main goals of this study were **(a)** to develop a virtual screening process to identify potential DPP-IV inhibitors of natural origin; **(b)** to evaluate the reliability of our virtual-screening protocol by experimentally testing the *in vitro* activity of selected natural-product hits; and **(c)** to use the most active hit for predicting derivatives with higher binding affinities for the DPP-IV binding site.

**Methodology/Principal Findings:**

We predicted that 446 out of the 89,165 molecules present in the natural products subset of the ZINC database would inhibit DPP-IV with good ADMET properties. Notably, when these 446 molecules were merged with 2,342 known DPP-IV inhibitors and the resulting set was classified into 50 clusters according to chemical similarity, there were 12 clusters that contained only natural products for which no DPP-IV inhibitory activity has been previously reported. Nine molecules from 7 of these 12 clusters were then selected for *in vitro* activity testing and 7 out of the 9 molecules were shown to inhibit DPP-IV (where the remaining two molecules could not be solubilized, preventing the evaluation of their DPP-IV inhibitory activity). Then, the hit with the highest activity was used as a lead compound in the prediction of more potent derivatives.

**Conclusions/Significance:**

We have demonstrated that our virtual-screening protocol was successful in identifying novel lead compounds for developing more potent DPP-IV inhibitors.

## Introduction

Type 2 diabetes mellitus (T2DM) is considered to be the “epidemic of the 21st century” and, consequently, the development of new therapies is one of the main challenges in drug discovery today [1]. While current T2DM therapies that increase insulin secretion have proven to have beneficial therapeutic effects, these treatments often suffer from undesirable side effects such as hypoglycemia and weight gain [2]. Therefore, there is a significant unmet medical need for better drugs to treat T2DM.

Recently, the inhibition of human dipeptidyl peptidase-IV (DPP-IV; EC 3.4.14.5) has emerged as a new treatment option for T2DM [3]. This enzyme belongs to the serine protease family and selectively removes N-terminal dipeptides from substrates containing proline or alanine as the second residue. The most important substrates of DPP-IV are incretins, such as glucagon-like peptide-1 (GLP-1) and glucose-dependent insulinotropic polypeptide (GIP) [4]. GLP-1 is released from intestinal L-cells in response to meals and performs the following actions: GLP-1 stimulates insulin biosynthesis and secretion, reduces glucagon release, slows gastric emptying, reduces appetite, and stimulates the regeneration and differentiation of islet B-cells [5]. Alternatively, GIP is produced by the duodenal K-cells and is extensively involved in glucose metabolism by enhancing insulin secretion [6]. Both peptides have very short half-lives (4 min for GIP and only 1–2 min for GLP-1) because of their rapid degradation by DPP-IV. Inhibiting DPP-IV prolongs the action of GLP-1 and GIP, which, in turn, improves glucose homeostasis with a lower risk of hypoglycemia. Consequently, DPP-IV inhibitors are of considerable interest to the pharmaceutical industry [7], and intense research activities in this area have resulted in the launch of sitagliptin, saxagliptin, alogliptin, linagliptin and vildagliptin to the market [8].

The DPP-IV binding site is highly druggable in the sense that tight and specific binding to the enzyme can be achieved with small molecules with drug-like physicochemical properties [9], [10]. The different interaction motifs used by these DPP-IV ligands include Ser630 (that together with Asp708 and His740 form the enzyme catalytic triad), the hydrophobic S1 pocket (formed by Tyr631, Val656, Trp659, Tyr662, Tyr666 and Val711), the hydrophobic S2 pocket (formed by Arg125, Phe357, Arg358, Tyr547, Pro550 and Asn710) and the N-terminal recognition region (formed by Glu205, Glu206 and Tyr662) [9], [11]. Based on the analysis of the DPP-IV crystal structures [12]–[18] and interpretation of the structure-activity relationship (SAR) data, both the lipophilic S1 pocket and the Glu205/Glu206 dyad can be considered as crucial molecular anchors for DPP-IV inhibition [9].

The large scaffold diversity and properties of natural products (NPs), such as structural complexity and drug similarity, makes these molecules ideal starting points for drug design. The main goal of this paper is to apply a virtual screening (VS) protocol to identify NPs with DPP-IV inhibitory activity as well as different scaffolds relative to known DPP-IV inhibitors that could be used as lead compounds in drug-design. In order to achieve this goal, we first identified complexes between DPP-IV and potent reversible inhibitors of non-peptide nature in the PDB. After validating the fit of the coordinates of binding site residues and inhibitors onto the corresponding electron density map, the validated DPP-IV complexes were overlapped to get the experimental poses of the inhibitor in the same orientation. Subsequently, the relative contribution of the different intermolecular interactions to the protein-ligand binding affinity was quantified to derive structure-based pharmacophores. The resulting energetically optimized pharmacophores were used to derive a structure-based common pharmacophore that contained key intermolecular interactions between DPP-IV and the inhibitors. The exclusion volumes were also determined and added to the pharmacophore. Then, the previous structure-based pharmacophore and a VS protocol were used to look for DPP-IV inhibitors in a NPs database [19], and the reliability of the prediction was demonstrated using *in vitro* testing to determine the DPP-IV inhibitory effects of representative VS hits. Lastly, the hit with the highest activity was used as a lead compound in a combinatorial screen for the prediction of more potent DPP-IV inhibitors.

## Results and Discussion

### Common Structure-based Pharmacophore Building and Description

**Figure 1:**
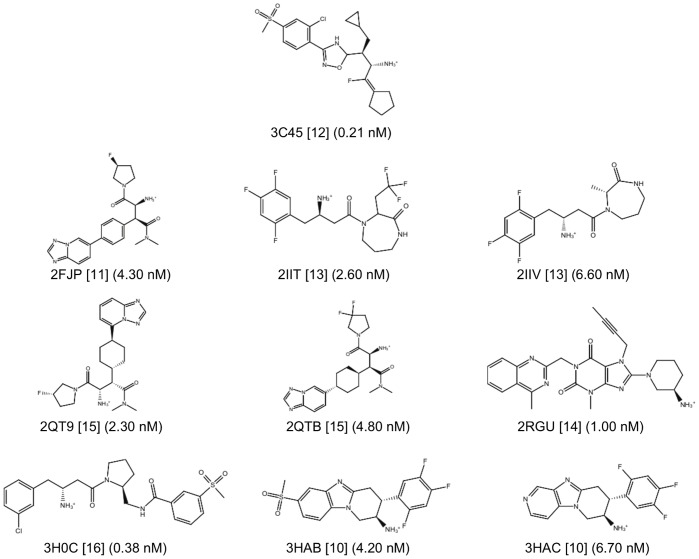
Drug-like reversible DPP-IV inhibitors used for the generation of the common structure-based pharmacophore with their corresponding IC_50_ values. The codes of the PDB complexes from which the ligand poses were used are also shown.

**Figure 2:**
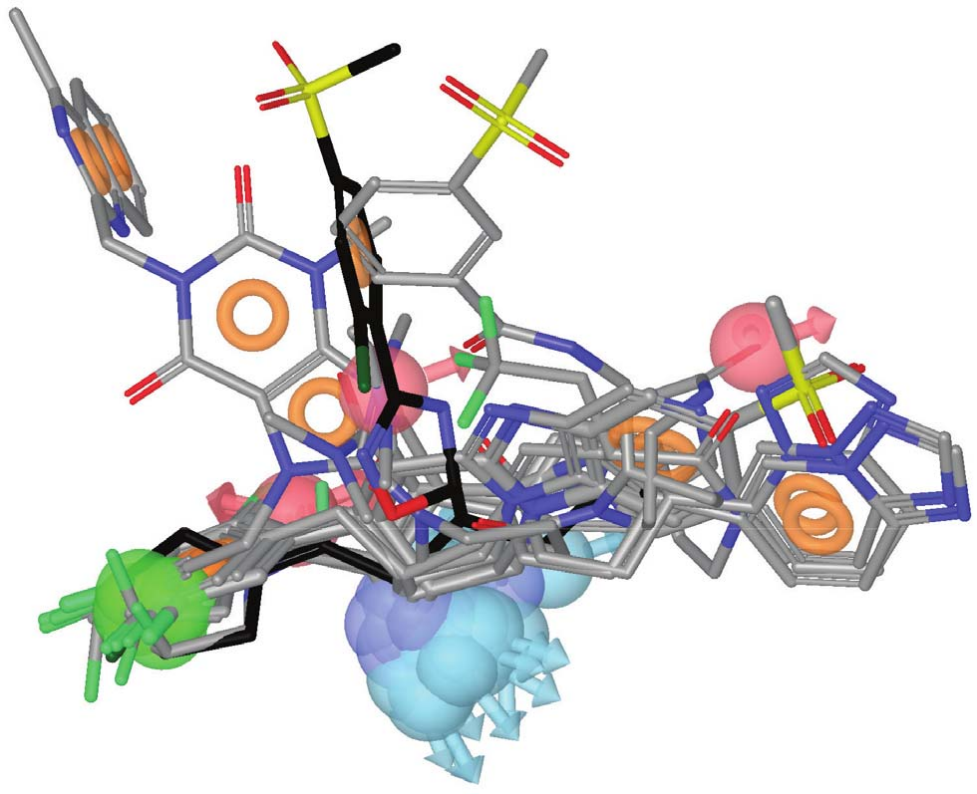
The relative location of the experimental poses of the ligands in **Figure 1** after DPP-IV superposition. The experimental pose for the most potent inhibitor (*i.e.*, the one at 3C45) is shown in black for reference. For each ligand, the energetically relevant pharmacophore sites are shown. Light red and light blue spheres represent the acceptor and donor features, respectively. The green spheres and orange torus display the hydrophobic regions and aromatic rings, respectively. Blue spheres represent positively charged regions.

**Figure 3:**
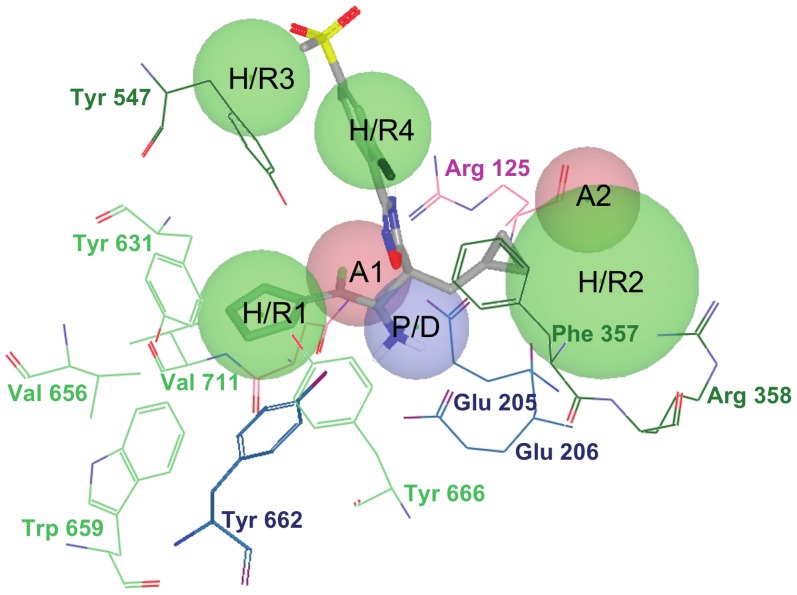
The structure-based common pharmacophore derived from the alignment of the poses in Figure 2 and shown in the context of the 3C45 active site. The pharmacophore is formed by two hydrogen-bond acceptors (*i.e.*, **A1** and **A2**), one positive/hydrogen-bond donor feature (*i.e.*, **P/D**) and 4 hydrophobic/aromatic ring sites (*i.e.*, **H/R1**, **H/R2**, **H/R3** and **H/R4**). The associated tolerances (*i.e.*, radii) of the pharmacophore are 1.8Å for **P/D**, **A1** and **A2**, 2.0Å for **H/R1**, **H/R3** and **H/R4** and 3.3Å for **H/R2**. Two out of these seven sites (*i.e.*, **P/D** and **H/R1**) are required during pharmacophore-based searches whereas the remaining five are optional. The **P/D** site interacts with the Glu205/Glu206 dyad whereas the **H/R1** site potentially fills the S1 pocket. The residues are colored according to the type of intermolecular interactions involved. For example, blue residues interact with donor sites, pink residues interact with acceptor sites and green residues are involved in hydrophobic contacts. Light green residues are a part of the S1 pocket.

There are currently 54 entries for DPP-IV in the Protein Data Bank (PDB; http://www.pdb.org; see Table 1) [20] but only 10 of those entries correspond to validated complexes of the native enzyme with potent reversible inhibitors of a non-peptide nature (see Figure 1). As a result, only these 10 entries are suitable for deriving reliable structure-based pharmacophores that capture the key intermolecular interactions needed for drugs to inhibit DPP-IV. In order to define a common background for DPP-IV inhibition, we identified features of inhibitors that make the most important contributions to the bioactivity of the ligand by first superposing all 10 PDB files. Then, the energetic pharmacophores were derived from the resulting coordinates, and energetically relevant pharmacophore sites were visually inspected for finding common or frequent ones. Figure 2 shows that all 10 pharmacophores have two sites in common (one positive/donor and one hydrophobic/aromatic ring) that often make the most important contribution to the protein-ligand binding affinity (see data for sites **P/D** and **H/R1** in Table 2). From these data, we inferred that these two sites are essential for the inhibition of DPP-IV and considered them to be required in the common structure-based pharmacophore (see Figure 3). Interestingly, previous studies have identified the lipophilic S1 pocket (formed by Tyr631, Val656, Trp659, Tyr662, Tyr666 and Val711) and the Glu205/Glu206 dyad as crucial molecular anchors for inhibition [9], [21], [22] and, in coherence with this, the mandatory hydrophobic/aromatic ring and positive/donor sites interact with the S1 pocket and Glu205/Glu206, respectively. Table 2 also shows that there are two other hydrogen-bond acceptors (**A1** and **A2**) and three hydrophobic/aromatic ring sites (**H/R2**, **H/R3** and **H/R4**) that, although not common to all experimental poses, could increase either protein-ligand binding affinity or drug-specificity. Moreover, it is remarkable that these sites correspond to interactions with other relevant areas from the DPP-IV binding site. For example, the **H/R2** site interacts with Phe357, Arg358 and Tyr547 in the S2 pocket (known to preferentially recognize large hydrophobic and aromatic side chains [11]). Therefore, these sites were also included as optional sites in the common structure-based pharmacophore (see Figure 3).

**Table 1:** Codes for DPP-IV structures currently available at PDB.

Valid PDB Structures			Discarted PDB Structures				
1N1M	2OPH	2RIP	(a)	(b)	(c)	(d)	(e)
**2FJP**	2OQI	3C43	1J2E	1TKR*	1R9N	1RWQ	1X70
2HHA	2OQV	**3C45**	1NU6	2AJL	1WCY	2BUB	2OAG
2I78	2P8S	3CCC	1NU8	2G5T	2BGN	2JID	3CCB
**2IIT**	2QJR	3D4L	1PFQ	2G5P	2BGR		3EIO
**2IIV**	2QOE	3F8S	1R9M	2G63			
2OGZ	**2QT9**	**3H0C**	1TK3	2I03			
2OLE	**2QTB**	**3HAB**	1U8E	2QKY			
2ONC	**2RGU**	**3HAC**	1W1I	3BJM*			

Some PDB structures were discarded for the following reasons: (a) the structures were of apo forms without inhibitor, (b) inhibitors were covalently linked with Ser630, (c) inhibitors were of oligopeptide nature, (d) there were no structural factors available in the PDB or (e) the scripts in the EDS failed to produce the map from the structural factors. PDB structures marked with an asterisk (*) have mutations in the enzyme to modify the activity. Only the PDB files from the “**Valid PDB Structures**” section with IC_50_ values ≤10 nM (in bold) were used to derive the corresponding structure-based common pharmacophore for DPP-IV inhibition (see Figure 1).

**Table 2:** Site contribution to the energy-optimized pharmacophores obtained from PDB complexes in bold from Table 1.

PDB	2FJP	2IIT	2IIV	2QT9	2QTB	2RGU	3C45	3H0C	3HAB	3HAC
**P/D**	−4,6	−4,13	−4,45	−4,09	−4,54	−1,66	−4,54	−4,81	−4,54	−4,29
**H/R1**	0,77	−1,29	−1,36	−1,25	−1,68	−0,075	−0,64	−1,18	−1,1	−1,25
*H/R2*	−0,69			−0,66	−0,68				−0,9	−0,69
*H/R3*						−1,94				
*H/R4*							−0,85			
H/R5						−0,56				
*A1*	−0,64			−0,4	−0,59					
*A2*			−0,62							
A3	−0,35				−0,35					
A4						−0,44				

Required and optional sites at the structure-based common pharmacophore are shown in bold and italics, respectively. The other sites are not part of the structure-based common pharmacophore. Data at the same raw for different PDB complexes indicate that the pharmacophore site is shared by these complexes.

### VS Workflow Description and Application to the NP Subset of the ZINC Database

**Figure 4:**
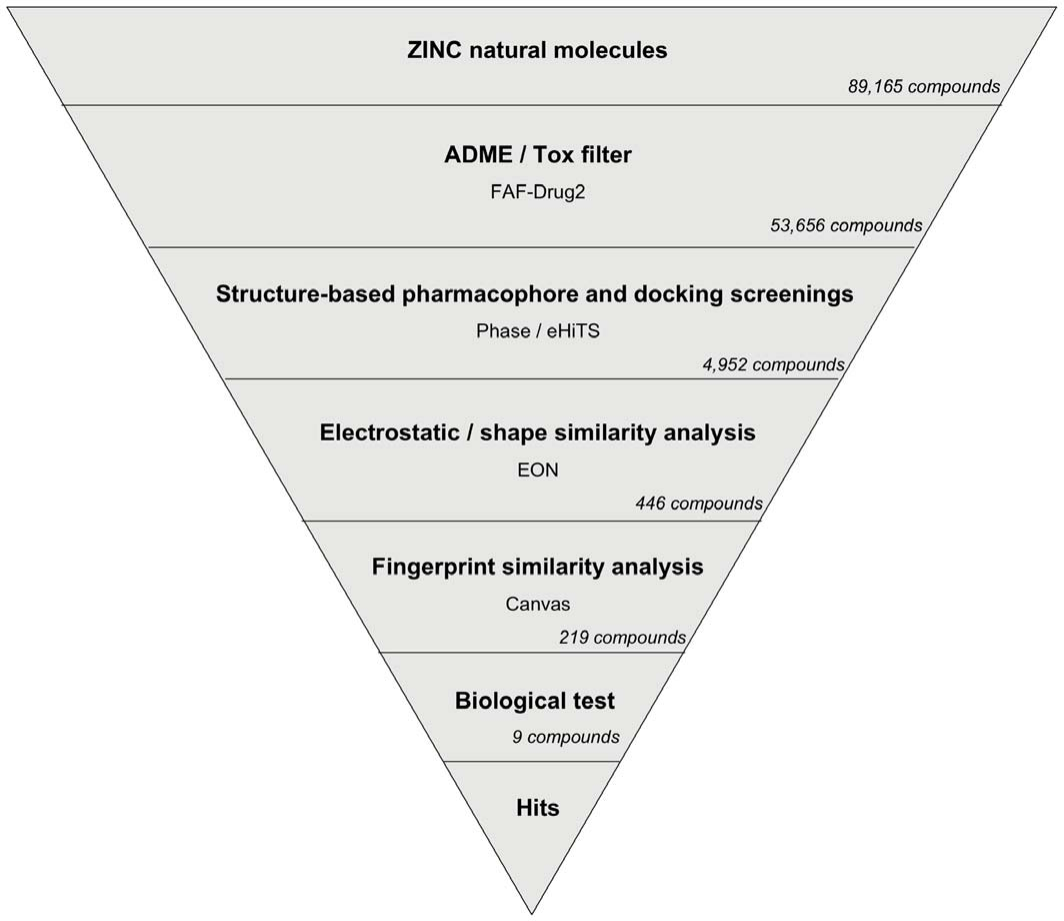
Schematic overview of the VS workflow and the procedure used for selecting the VS hits that were tested for DPP-IV inhibitory activity. For the VS, the number of compounds that passed each step and the programs used are showed. For the selection of VS hits for bioactivity testing, the numbers show either how many VS hits are scaffold-hopping candidates for DPP-IV inhibition (*Fingerprint similarity analysis* step) or how many molecules were experimentally tested for bioactivity (*Biological test* step).

The VS workflow (see Figure 4) consisted of several sequential steps where the output molecules of one step were the input molecules for the next step and so on. The NP subset of the ZINC database was used as the source of molecules to which our VS schema was applied to search for new DPP-IV inhibitors. Initially, these 89,165 molecules were submitted to an ADME/Tox filter with the FAF-Drugs2 tool [23] aimed at discarding molecules that were either potentially toxic or exhibited poor ADME properties.

Conformers for molecules with appropriate ADME/Tox properties were then filtered with Phase through the structure-based common pharmacophore. Ligands with at least one hit in the Phase search were then used in a protein-ligand rigid-docking study and docked onto the ligand binding site of the DPP-IV conformation present in the 3C45 PDB file [14]. In order to find docking poses that were compatible with the pharmacophore, the resulting ligand poses were filtered again with Phase through the structure-based common pharmacophore using the same filtering conditions as in the first Phase run but without reorienting the poses (*i.e.*, the score in place option was used). From these two pharmacophore screens, we obtained 4,952 compounds (see Figure 4) with at least one pose that was both compatible with the DPP-IV active site and had functional groups that match the 3D location of the two compulsory sites and at least one of the optional sites of the structure-based common pharmacophore.

Finally, the poses for the 4,952 compounds from the second pharmacophore screen were submitted to a shape and electrostatic-potential comparison with the experimental pose of the DPP-IV inhibitor at the PDB file 3C45 (that has the smallest IC_50_ for all the non-peptide reversible inhibitors found in DPP-IV-inhibitor complexes at the PDB [14]; see Figure 1). The shape and electrostatic-potential comparison identified 446 hit molecules with potential DPP-IV inhibitory activity (see Figure 4).

### Finding New Scaffolds of Natural Origin for DPP-IV Inhibitors

**Figure 5:**
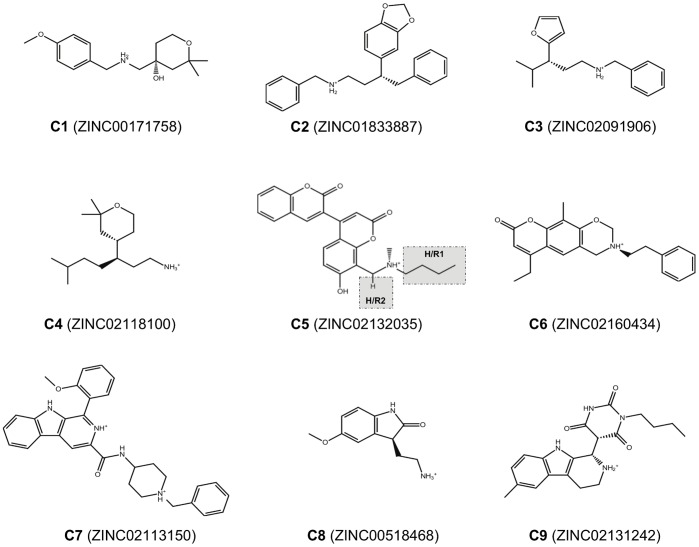
Chemical structures and ZINC codes for the 9 molecules selected for experimentally testing whether these compounds exhibited DPP-IV inhibitory activity. The insolubility of **C4** and **C6** prevented these compounds from being assayed for DPP-IV inhibitory activity. Positions in the **C5** structure that will be replaced by substituents to identify derivatives with higher binding affinity on the DPP-IV binding site are **(a)** indicated with a grey background and **(b)** annotated with the label of the corresponding site in the common structure-based pharmacophore (see Figure 3).

**Figure 6:**
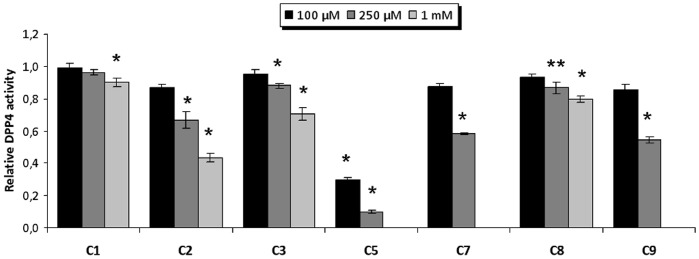
Dose-response results for the *in vitro* inhibition of DPP-IV by C1, C2, C3, C5, C7, C8 and C9. The relative DPP-IV inhibitory activity with or without the selected NPs (vehicle, 1% DMSO) is shown where each column represents the average ± SEM (n = 3 or 4). The insolubility of **C5**, **C7** and **C9** in DMSO at 1 mM prevented the measurement of DPP-IV inhibitory activity. *p<0.05 **p<0.1 vs vehicle, T-student.

**Figure 7:**
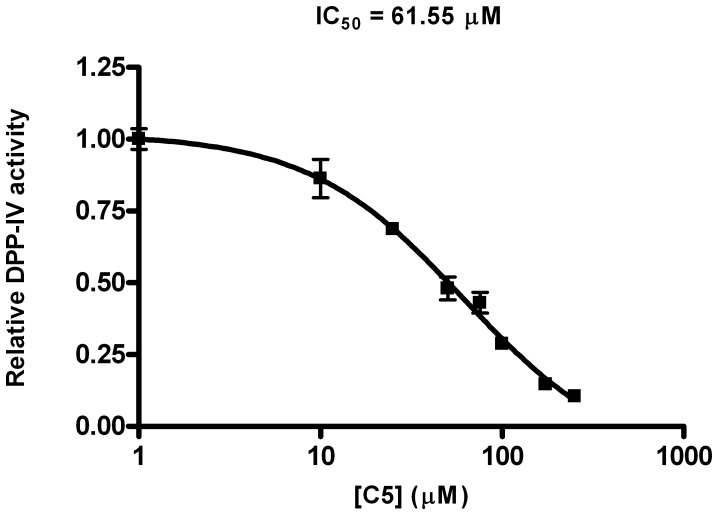
DPP-IV inhibitory dose-response curve obtained for C5 via a competitive binding assay.

One of the most important challenges of any VS workflow is the ability to find molecules with the required activity but without trivial similarity (in terms of chemical structure) to known active compounds. To determine which of the 446 potential DPP-IV inhibitors predicted by our VS workflow could be considered as new lead molecules, we merged the 446 potential DPP-IV inhibitors with 2,342 known DPP-IV inhibitors that were obtained from the BindingDB database [24]. After calculating the 2D fingerprints of these inhibitors, the resulting set was classified into 50 clusters by means of a hierarchical cluster analysis (data not shown). Notably, 12 out of the 50 clusters obtained consisted exclusively of NPs that were previously unidentified as DPP-IV inhibitors. The 219 molecules that belong to these 12 clusters are scaffold-hopping candidates for DPP-IV inhibition (see Table S1). To prove the reliability of our predictions, we selected 9 molecules (**C1** and **C2** from cluster 30, **C3** from cluster 36, **C4** from cluster 37, **C5** and **C6** from cluster 41, **C7** from cluster 45, **C8** from cluster 49 and **C9** from cluster 50) from 7 of these 12 clusters (see Figure 5) and tested their effects on the DPP-IV activity using an *in vitro* assay. The results of this experiment demonstrated that 7 out of the 9 molecules (**C1**, **C2**, **C3**, **C5**, **C7**, **C8** and **C9**) inhibit DPP-IV (see Figure 6). The remaining molecules, **C4** and **C6**, could not be solubilized, preventing the evaluation of their DPP-IV inhibitory activity. The lack of DPP-IV inhibitory activity for **C5**, **C7** and **C9** at 1mM was also due to insolubility (see Figure 6). Furthermore, Figure 6 shows that from all the tested molecules, **C5** is the most potent inhibitor with an IC_50_ of 61.55 µM (see Figure 7). With the exception of **C1**, which significantly inhibited DPP-IV only at 1 mM, the rest of the molecules significantly inhibit DPP-IV at 0.25 mM (see Figure 6) showing a dose-response effect. Moreover, a SciFinder search (Chemical Abstracts Service, Columbus, Ohio, USA; http://www.cas.org/products/sfacad) of the literature revealed that none of these 7 molecules have been reported as antidiabetic drugs. In fact, no bioactivity has been described for these 7 molecules.

### Structural Analysis of the Inhibition of DPP-IV by C1, C2, C3, C5, C7, C8 and C9

**Figure 8:**
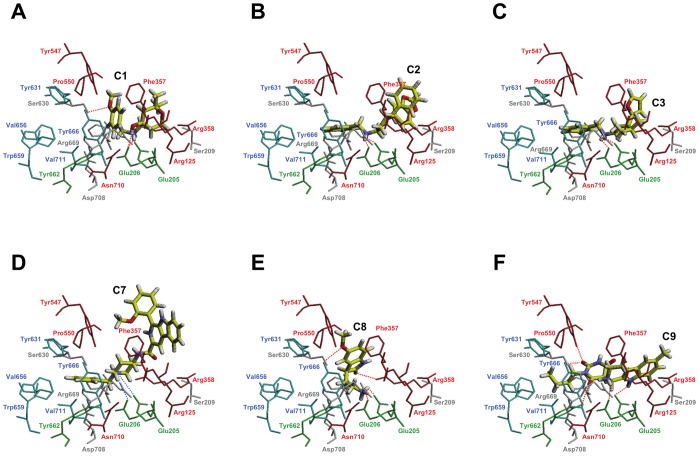
Docking poses for C1, C2, C3, C7, C8 and C9 at the 3C45 binding site. All of the panels in this figure and in Figure 9 are in the same relative orientation to allow for easier comparisons between the predicted poses. Residues at the DPP-IV binding site are colored according to the subsite where they belong (*i.e.*, residues from the S1 pocket are colored in cyan, those from the S2 pocket are red and those from the N-terminal recognition region are green). Other important residues that have not been classified in any pocket are colored in white. Dashed lines are used to show intermolecular hydrogen bonds (in red) or charge-charge interactions (in blue).

**Figure 9:**
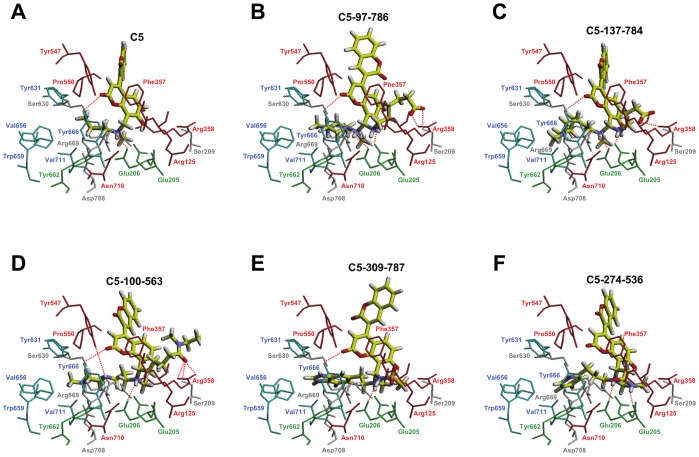
Docked poses for C5 (panel A) and the five C5 derivatives with the highest predicted affinities (panels from B to F) at the 3C45 binding site. All of the panels in this figure and in Figure 8 are in the same relative orientation to allow for easier comparisons between the predicted poses. Residues at the DPP-IV binding site are colored by the same criteria described in Figure 8. Dashed lines are used to show intermolecular hydrogen bonds.

The docking of **C1**, **C2**, **C3**, **C5**, **C7**, **C8** and **C9** in the DPP-IV binding site of the 3C45 structure demonstrated that these molecules match the structure-based common pharmacophore in the same orientation, sharing the same intermolecular interactions with DPP-IV (see Figures 8 and 9A). With the exception of **C7** in which the positive charge of the tertiary amine forms a salt bridge with Glu205/Glu206 (see Figure 8D), all compounds use primary or secondary amines to form hydrogen bond interactions with either Glu206 or with the Glu205/Glu206 dyad side chains (see Figures 8 and 9A). Additionally, all molecules filled the S1 pocket (partially in the case of **C1** and **C8,** which could explain why these two molecules have lower activities as DPP-IV inhibitors; see Figure 6) establishing one intermolecular interaction that corresponds to the compulsory **H/R1** site of our common structure-based pharmacophore (see Figure 3). Moreover, it is worthwhile to mention that some molecules could potentially form additional hydrogen bonds with DPP-IV. For example, the hydroxyl and the methoxy groups of **C1** could hydrogen bond with the side chains of Glu206 and Ser630, respectively (see Figure 8A). **C8** forms two additional hydrogen bonds with the side chains of Arg358 and Tyr666 (see Figure 8E). Finally, **C9** could form three additional hydrogen bonds with the side chains of Tyr547, Ser630 and Tyr662 (see Figure 8F).

Figure 9A shows the best docking pose of **C5** in the DPP-IV binding pocket where its tertiary amine hydrogen bonds with Glu206. The carbonyl oxygen of the 7-hydroxy-2H-chromen-2-one moiety could also hydrogen bond with the Tyr666 side chain. The S1 pocket is occupied by the **C5** butyl chain that could form hydrophobic interactions with Tyr662, Tyr666 and Val711. Finally, the chromene ring of the 7-hydroxy-2H-chromen-2-one moiety forms π-π interactions with Phe357. Interestingly, this interaction with Phe357 has been shown to be directly related to the increased potency of synthetic DPP-IV inhibitors relative to those that lack this interaction [13], [15], [25]–[27]. Therefore, the fact that this interaction is only present at **C5** (see Figures 8 and 9A) would explain why this molecule shows higher bioactivity than the other compounds assayed (see Figure 6). Moreover, an electrostatic and shape comparison of the 7 poses in Figures 8 and 9A revealed that the molecule with the highest similarity to the 3C45 ligand (with the lowest IC_50_; see Figure 1) is **C5** (results not shown). The ET_combo score for this comparison is 1.050, which corresponds to a shape and electrostatic contribution of 0.628 and 0.422, respectively. Remarkably, the same analysis with **C2** (which shows a significant bioactivity as DPP-IV inhibitor; see Figure 6), also has a significant ET_combo score of 1.038.

### Predicting ZINC02132035 Derivatives with Higher Binding Affinity on DPP-IV

Although none of the seven *in vitro* assayed VS hits showed activity in the nanomolar range, these hits incorporate scaffolds with no previously described effects on DPP-IV activity and, consequently, are of interest from a medicinal chemistry point of view as lead compounds for more potent DPP-IV inhibitors. With this goal in mind, we have predicted derivatives for the most potent DPP-IV inhibitor found in our dose-response studies (*i.e.*, **C5;** see Figure 6) by **(1)** using a fragment-based docking approach to identify which intermolecular interactions with the DPP-IV binding site could improve the binding affinity of **C5** derivatives relative to **C5**; **(2)** using this information to identify where changes in **C5** should be made; and **(3)** accordingly building **C5** derivatives and predicting their relative binding affinities.

The comparison of the XP descriptors from **C5** and from the docked poses of the fragments showed that while some of the terms of the scoring function are 0.00 Kcal/mol for **C5**, their corresponding value for 13 out of 50 fragments is in the [-2.48, -0.83] Kcal/mol range (see Table S2). Interestingly, 12 out of 13 of these fragments bind at the locations of three of the sites of our structure-based common pharmacophore (*i.e.*, **H/R1**, **H/R2** and **H/R4**), whereas the remaining fragment is close to the **H/R1** site (see Table S2). These findings demonstrate that our pharmacophore is able to *capture* all of the essential features for an inhibitor interaction with the DPP-IV binding-site, which would explain why all of the assayed molecules show activity as DPP-IV inhibitors (see Figure 6). Moreover, the analysis in Table S2 shows that **C5** activity can be improved if **(1)** its butyl group matching the **H/R1** site is replaced by a group that is able to interact with the lipophilic atoms of the S1 pocket either by producing the so-called *hydrophobic enclosure reward* (that would consist of enclosing the two sides of the substituent –at a 180° angle– on the hydrophobic environment of the S1 pocket) or by making π-cation interactions with the aromatic side chains in this pocket and **(2)** groups that match the **H/R2** site (optional in our pharmacophore but present in half of the ligands used to derive the pharmacophore; see Table 2) and that are able to make hydrophobically packed correlated H-bonds are added to **C5**.

The substituents that were attached to the **C5** core to obtain the top five derivatives with the highest predicted binding affinity for the DPP-IV binding site are shown in Table S3. None of the five molecules are currently registered in ChemSpider (http://www.chemspider.com), whereas their analysis with FAF-Drugs2 shows that all of these derivatives exhibit the proper ADMET properties. Therefore, these derivatives are undescribed drug-like molecules that, according to their XP GScores (see Table S3), would show a significant increase in their binding affinity relative to **C5** (*i.e.*, -4.2 Kcal/mol).

Figure 9 shows the docked poses for **C5** derivatives compared with **C5** and can be used to explain the structural basis of the expected increase in binding affinity. Remarkably, the XP GScores for these poses are in the -9.5 to -11.8 Kcal/mol range (see Table S3), whereas the GScores for the experimental poses of the DPP-IV inhibitors shown in Figure 1 are in the -5.8 to -11.0 Kcal/mol range (results not shown). Therefore, the **C5** derivatives reported in Table S3 are likely to exhibit nanomolar activity as DPP-IV inhibitors.

As shown in Figure 9, the **C5** derivatives usually maintained the most important protein-ligand interactions found for the **C5** core. Moreover, Table S3 also shows that all of the substituents that have replaced the original **C5** butyl group (*i.e.*, at the **H/R1** site) have a common positive formal charge that, according to results shown in Figure 9, allows them to form π-cation interactions with two of the aromatic residues in the S1 pocket (*i.e.*, Tyr662 and Tyr666). Additionally, some of the substituents at this location (*i.e.*, **97** in **C5-97-786**, **100** in **C5-100-563** and **274** in **C5-274-536**; see Table S3) also aid in increasing the protein-ligand binding affinity by enclosing the two sides of the corresponding ring in the lipophilic protein environment in the S1 pocket (results not shown). Furthermore, all substituents at the **H/R2** site (except the one in **C5-309-787**) are able to make hydrogen bonds either with the S2 pocket residue Arg358 (*i.e.*, **786** in **C5-97-786**, **784** in **C5-137-784** and **563** in **C5-100-563**; see Figures 9B, 9C and 9D) or with Arg669 (*i.e.*, **536** in **C5-274-536**; see Figures 9F). The **786** substituent in **C5-97-786** is also able to make a hydrogen bond with the Ser209 side chain (see Figure 9B). Remarkably, there are SAR studies with a structurally distinct series of DPP-IV inhibitors that show **(1)** a 4-fold loss of potency when substituents that interact with the side chains of Ser209 and Arg358 are removed [28], **(2)** a 2-fold increase in inhibition when a carboxylic acid that interacts with Arg358 is introduced [27], and **(3)** a 6-fold increase in inhibition when a 3-pyridyl group that interacts with Ser209 is introduced [29]. Therefore, the substituents selected for the **H/R2** site by the combinatorial screen are able to form the intermolecular interactions with the S2 pocket that previous SAR studies with anti-diabetic drugs have shown to increase the affinity for the DPP-IV binding site.

### Conclusions

The challenge of any VS protocol consists of using *in silico* tools to predict which molecules in a database have the required activity against a specific target. The results of the present study demonstrate that our VS protocol is highly successful in the non-trivial identification of DPP-IV inhibitors with no chemical-structure similarities to known activities. Therefore, scaffold hopping on this target can be achieved. Moreover, this is the first time that anti-diabetic activity has been described for **C1** (*i.e.*, ZINC00171758), **C2** (*i.e.*, ZINC01833887), **C3** (*i.e.*, ZINC02091906), **C5** (*i.e.*, ZINC02132035), **C7** (*i.e.*, ZINC02113150), **C8** (*i.e.*, ZINC00518468) and **C9** (*i.e.*, ZINC02131242).

Although the IC_50_ of the 7 hit molecules indicates their *in vitro* activity is significantly lower than that of most known DPP-IV inhibitors used to derive the structure-based common pharmacophore (see Figure 1), it is important to remark that these molecules can be used as lead compounds for developing more potent inhibitors by means of SAR studies. Furthermore, these 7 molecules were selected based on their commercial availability, cost and purity with the primary goal of testing the performance of our VS protocol. Therefore, it is possible that there are other molecules among the remaining 210 molecules in clusters 10, 29, 30, 36, 37, 38, 40, 41, 44, 45, 49 and 50 (see Table S1) that could be better starting points than **C5** for the rational drug design of potent and selective DPP-IV inhibitors with new chemical scaffolds. Remarkably, our work makes a significant contribution to the discovery of DPP-IV inhibitors of natural origin (described, at present, for only few NPs [21], [30]–[32]) from a quantitative point of view. Moreover, this work is also applicable to screen synthetic molecules databases when looking for antidiabetic activity.

Finally, we would like to note the high degree of agreement between our predictions (without making any prior knowledge-based assumptions that could bias our decisions) about the derivatization of **C5** to increase the binding affinity (*e.g.*, introducing side chains that could interact with Ser209 and Arg358) and what SAR studies have reported in the literature for achieving this increase. Therefore, this strongly supports the reliability of our combinatorial screening results.

## Methods

### Criteria for Selecting the 3D Structures for DPP-IV Complexes used to Derive the Common Structure-based Pharmacophore

Coordinates for complexes between DPP-IV and potent reversible inhibitors were obtained from the PDB with the help of the following information: (a) LigPlot [33] schemes downloaded from the PDBsum website (http://www.ebi.ac.uk/pdbsum/) that were used to confirm the non-peptide and reversible character of the DPP-IV inhibitor present in each complex and; (b) IC_50_ values directly extracted from the literature describing the complexes (only complexes with inhibitors with IC_50_≤10 nM were considered). Furthermore, the complexes with at least one mutation in their amino acid sequences were discarded. The reliability of the binding-site residues and inhibitor coordinates was assessed for the remaining complexes by visually inspecting their degree of fitness to the corresponding electron density map available from the Uppsala Electron Density Server (EDS; http://eds.bmc.uu.se/eds/) [34].

### Superposition of the Selected DPP-IV Structures

The coordinates from the PDB complexes that met all the mentioned requirements were superposed with the DeepView v3.7 program (http://spdbv.vital-it.ch/) [35] to have the complexes in the same relative orientation. Only the resulting re-oriented coordinates for these PDB files were used during the subsequent structure-based pharmacophore generation and in the steps of the VS workflow (*i.e.*, pharmacophore-based searches, protein-ligand docking studies and shape and electrostatic-potential comparisons) where spatial orientation is crucial.

### Common Structure-based Pharmacophore for DPP-IV Inhibition

Energetic structure-based pharmacophores were built from the superposed coordinates of the previously selected complexes by means of the Glide-based procedure developed by Schrödinger (Schrödinger LLC., Portland, USA; http://www.schrodinger.com) [36]. According to this procedure, pharmacophore sites are ranked based on the Glide XP energies with the advantage that each contribution to the protein-ligand interactions is quantified. Therefore, energetically favorable features can be incorporated into the pharmacophore with preference over energetically weaker features. The resulting individual energetic pharmacophores were used for the construction of a common structure-based pharmacophore for DPP-IV reversible inhibition. This pharmacophore consists on two compulsory sites (one positive/donor and one hydrophobic/aromatic ring) whereas the remaining acceptor and hydrophobic/aromatic ring sites are optional. The associated tolerances for the different sites are 1.8Å for **P/D**, **A1** and **A2**, 2.0Å for **H/R1**, **H/R3** and **H/R4** and 3.3Å for **H/R2**. The pharmacophore was completed with receptor-based excluded volumes that schematically represent the location of the DPP-IV residues that form the binding pocket by applying the ***Receptor-Based Excluded Volumes*** graphic front-end from Phase v3.1 (Schrödinger LLC., Portland, USA; http://www.schrodinger.com) [37] to the PDB file 3C45. The ***Sphere filters*** parameter values were set to the following criteria: (a) ignoring receptor atoms whose surfaces were within 0.25 Å of ligand surface; and (b) limit excluded volume shell thickness to 10 Å. Otherwise, the remaining parameter values used were the default values.

### Ligand Selection for VS Purposes

Ligands for VS purposes were downloaded from the Natural Products subset of the ZINC database (http://wiki.bkslab.org/index.php/Natural_products_database) [19]. This dataset contains 89,165 commercially available natural products and natural-product derivatives, making the dataset suitable for experimentally testing the success of a VS workflow.

### ADME/Tox Filter

The ADME/Tox filter was carried out with the FAF-Drugs2 tool [23]. The drug-like properties of a compound were evaluated using the Lipinski rule [38]. The Lipinski rule is based on a set of property values, such as the number of hydrogen-bond donors and acceptors, the molecular weight and the logP, that were derived from drugs with good ADME characteristics. Molecules that adhere to the Lipinski rule are expected to be active in humans after oral admission. Only one violation of this rule was allowed. Molecules containing toxic groups were filtered using the 204 substructures for “warhead” chelators, frequent hitters, promiscuous inhibitors and other undesirable functional groups available in the FAF-Drugs2 tool [23].

### Ligand Setup

The 3D structures of the ligands for VS purposes were incorporated into LigPrep v2.3 (Schrödinger LLC., Portland, USA; http://www.schrodinger.com) and improved by cleaning. The cleaning process was carried out using the following parameters: (a) the force field used was OPLS 2005; (b) all possible ionization states at pH 7.0±2.0 were generated with Ionizer; (c) the desalt option was activated; (d) tautomers were generated for all ionization states at pH 7.0±2.0; (e) chiralities were determined from the 3D structure; and (f) one low-energy ring conformation per ligand was generated. Conformations and sites for the resulting ligand structures were determined during the generation of the corresponding Phase [37] databases with the ***Generate Phase Database*** graphic front-end. Default parameter values were used during this conformer generation with the exception of the maximum number of conformers per structure, which increased from 100 (the default value) to 200. The conformer sites were generated with definitions made by adding the ability to consider aromatic rings as hydrophobic groups to the default built-in Phase definitions.

### Structure-based Pharmacophore Screening

The initial filtering through the structure-based common pharmacophore was performed with Phase v3.1 using the following steps: (a) search in the conformers database, (b) do not score in place the conformers into the structure-based common pharmacophore (*i.e.*, allow reorientation of the conformers to determine if they match the pharmacophore or not), (c) match the two compulsory sites of the structure-based common pharmacophore and at least one of the optional sites, (d) do not have a preference for partial matches involving more sites and (e) use the excluded volumes from the structure-based common pharmacophore. Default values were used for the rest of the options and parameter values used during this search. For the second pharmacophore screening, the same filtering options of the first pharmacophore matching were applied with the exception that now no re-orientation of the poses was allowed during the search (*i.e.*, the *score in place* option was used) because it was performed by using docked poses.

### Protein-ligand Docking during the VS

During the VS, the protein-ligand docking was performed with eHiTS v2009 (SimBioSys Inc., Toronto, Canada; http://www.simbiosys.ca/ehits) [39], and ligands were docked into the ligand binding site of the DPP-IV conformation present in the 3C45 PDB file [14]. The receptor was considered to be a rigid body and the ligands as flexible such that free rotation was allowed around the single bonds of the ligand. Default docking conditions were selected with the exception of the size of the sides of the cubic box encompassing the DPP-IV binding site, which was increased from 10 Å to 15 Å.

### Electrostatic and Shape Similarity Screening

The software EON v2.0.1 (OpenEye Scientific Software, Inc., Santa Fe, New Mexico, USA; http://www.eyesopen.com) determines the electrostatic potentials of two compounds and consequently calculates the Electrostatic Tanimoto combo score (ET_combo). The ET_combo is the sum of the Shape Tanimoto (ST) and the Poisson-Boltzman Electrostatic Tanimoto scores. The Shape Tanimoto (ST) score is a quantitative measure of three-dimensional overlap where 1 corresponds to a perfect overlap (*i.e.*, the same shape) [40]. The Poisson-Boltzman Electrostatic Tanimoto score (ET_pb) compares the electrostatic potential of two small molecules where 1 corresponds to identical potentials and negative values correspond to the overlap of positive and negative charges [41]. Only those molecules that have both ET_pb and ST score values higher than 0.623 and 0.244, respectively, were selected and visualized with VIDA v4.0.3 (OpenEye Scientific Software, Inc., Santa Fe, New Mexico, USA; http://www.eyesopen.com). These threshold values were chosen after analyzing which ET_pb and ST score values are obtained when the DPP-IV inhibitor in PDB file 3C45 is compared with the experimental poses of the rest of the inhibitors from which the common pharmacophore was derived (see Figure 1).

### Hit Selection for Further Experimental Assays on DPP-IV Activity

The molecules that survived the electrostatics/shape similarity filter were merged with 2,342 known inhibitors obtained from the BindingDB database [24], and then clustered using Canvas v1.2 (Schrödinger LLC., Portland, USA; http://www.schrodinger.com). MOLPRINT2D fingerprints [42], using a fingerprint precision of 32 bits, were calculated for each molecule and then hierarchical clustering, based on Tanimoto similarities, was performed resulting in 50 clusters. Nine compounds from 7 of the 12 clusters exclusively formed by NPs that were previously unidentified as DPP-IV inhibitors were selected based on their commercial availability, cost and purity (≥92%) for *in vitro* assays of DPP-IV inhibitory activity. These compounds were ZINC00171758 (*i.e.*, **C1**), ZINC01833887 (*i.e.*, **C2**), ZINC02091906 (*i.e.*, **C3**), ZINC02118100 (*i.e.*, **C4**), ZINC02132035 (*i.e.*, **C5**) and ZINC02160434 (*i.e.*, **C6**), ZINC02113150 (*i.e.*, **C7**), ZINC00518468 (*i.e.*, **C8**) and ZINC02131242 (*i.e.*, **C9**), which were all purchased from InterBioScreen, Ltd (http://www.ibscreen.com).

### *In vitro* Assay of the Effect of Selected Compounds on the DPP-IV Activity

The *DPP-IV Drug Discovery Kit*-*AK499* (Enzo Life Sciences International, Inc.) was used to conduct DPP-IV inhibition assays. Briefly, 10 µL of each compound were added to commercial recombinant human DPP-IV. Stock solutions of the assayed compound were made in DMSO and diluted in buffer (50 mM Tris-HCl) to final concentrations ranging from 10–1000 µM in the assay. The final concentration of DMSO in the assay was 1%. After 10 minutes of incubation at 37°C, the reaction was initiated by the addition of the fluorimetric substrate H-Gly-Pro-AMC. Fluorescence was measured continuously for 30 minutes at Ex: 380 nm/Em: 460 nm in a Biotek FLx800 Fluorescence Microplate Reader. At least three independent assays were performed, each with two technical replicates. A standard DPP-IV inhibitor (P32/98 from Biomol, Germany) served as positive control.

### IC_50_ Calculation

IC_50_ was determined using GraphPad Prism v4.0 for Windows (GraphPad Software, San Diego CA, USA; http://www.graphpad.com) by fitting the experimental data from the *in vitro* assay to a nonlinear regression function using a four-parameter logistic equation.

### Docking of Novel DPP-IV Ligands

Docking studies of DPP-IV inhibitors **C1**, **C2**, **C3**, **C5**, **C7**, **C8** and **C9** were performed with the software Glide v5.6 (Schrödinger LLC., Portland, USA; http://www.schrodinger.com) using the DPP-IV coordinates that can be found using the 3C45 PDB code. The binding site was defined using the default options of the *Receptor Grid Generation* panel. Standard-precision (SP) docking was initially used to screen the ligands. The flexible docking mode was selected such that Glide internally generated conformations during the docking process. No constraints were selected for docking. Each docking run recorded at most ten poses per ligand that survived the post-docking minimization. The best docking poses for the novel DPP-IV ligands were selected by not only considering the docking scores but also by taking into account the results of the visual inspection of all docking poses. This visualization was performed with Maestro v9.2 (Schrödinger LLC., Portland, USA; http://www.schrodinger.com). Further, the location of the selected poses within the binding site was refined with extra-precision (XP) to maximize the intermolecular interactions between **C1**, **C2**, **C3**, **C5**, **C7**, **C8** and **C9** and the DPP-IV binding site. The resulting **C5** docked pose was subsequently used for lead-optimization.

### Lead-optimization from the Most Active Compound

Improvement of the binding affinity of **C5** was performed in two steps. Initially, a library formed by 50 fragments (and available with the last version of the Schrödinger suite) was docked at the 3C45 binding site using the Glide XP mode. Then, the XP visualizer tool (Schrödinger LLC., Portland, USA; http://www.schrodinger.com) was used to compare the values for the different XP descriptors between the **C5** docked pose and the highest score pose for each fragment. We focused the comparisons on XP descriptors that have no contributions to the XP GScore of **C5** but instead show significant values for some fragments (*i.e.*, the **PhobEn**, **PhobEnHB**, **PhobEnPairHB** and **πCat** descriptors; see Table S2). This comparison resulted in potential attachment positions of **C5** for testing substituents that could improve the DPP-IV inhibitory activity by increasing the corresponding affinity for the target.

The substituents available in the CombiGlide Diverse Side-chain Collection v1.2 (which contains all reasonable ionization and tautomeric states for a collection of 817 representative functional groups commonly found in pharmaceuticals, with linkers of variable lengths) were used to replace the original substituents of **C5** at each attachment point (see Figure 5). This replacement was carried out using the Virtual Combinatorial Screening workflow available in CombiGlide v2.7 (Schrödinger LLC., Portland, USA; http://www.schrodinger.com). During the docking step of this workflow, docked poses were restricted to be within a maximum RMSD of 1.0 Å relative to the **C5** core in the **C5** predicted pose (see Figure 9A). Moreover, those **C5** derivatives resulting from a single substitution at any position on the core structure were docked, and those reagents at each position that did not seem promising were screened out. This elimination significantly reduced the number of fully substituted structures to be docked. The remaining options during the combinatorial screening were set by default. Finally, the top 100 scored poses for the **C5** derivatives were selected for refinement with Glide XP using the default options, and the resulting top-five ranked poses were chosen for further analyses (see Table S3).

## Supporting Information

Table S1 **Predicted scaffold-hopping candidates for DPP-IV inhibition.** This table shows ZINC codes for the 219 hit molecules predicted to inhibit DPP-IV that belong exclusively to clusters containing NPs that were previously unidentified as DPP-IV inhibitors. The best results of the shape and electrostatic-potential comparisons for each hit molecule with the ligand of 3C45 crystallized structure are shown. The Tanimoto values for the comparison between the electrostatic potentials of the molecules (using an outer dielectric of 80) are shown in the ET_PB columns. Furthermore, the values for the comparison between shapes are shown in the ET_Shape columns. The sum of the ET_PB and ET_Shape values is reported in the Combo columns. Hits from each cluster are sorted according to their decreasing combo value. ZINC00171758 and ZINC01833887 (cluster 30), ZINC02091906 (cluster 36), ZINC02118100 (cluster 37), ZINC02132035 and ZINC02160434 (cluster 41), ZINC02113150 (from cluster 45), ZINC00518468 (cluster 49) and ZINC02131242 (cluster 50) were tested in an *in vitro* assay to validate the success rate of our predictions (in bold in Table S1). Due to the insolubility, ZINC02118100 (cluster 37) and ZINC02160434 (cluster 41) could not be tested.
(PDF)

Table S2 **Docked fragments that have significant contributions to the GScore for XP descriptors that are 0.00 Kcal/mol for C5.** The most potent DPP-IV inhibitor found by our dose-response studies (*i.e.*, **C5**) has no contribution to the GScore by the following XP descriptors: **(a)** PhobEn (*i.e.*, hydrophobic enclosure reward); **(b)** PhobEnHB (*i.e.*, reward for hydrophobically packed H-bond); **(c)** PhobEnPairHB (*i.e.*, reward for hydrophobically packed correlated H-bond); and **(d)** πCat (*i.e.*, reward for π-cation interactions). This table displays the docked fragments showing the highest values for these XP descriptors and the common pharmacophore sites of Figure 3 that are matched to the corresponding fragment, if any.
(DOC)

Table S3 **Top five C5 derivatives according to their XP GScores. The top five C5 derivatives according to their XP GScore values.** The structures of the substituents that were attached to the **C5** core at the two replacement sites (see Figure 5) are shown. The code for each molecule is obtained by adding the CombiGlide Diverse Side-chain Collection code for the substituents at the H/R1 and at the H/R2 sites to **C5**.
(DOC)
