# Supplementary material for: Identification of Novel Human Dipeptidyl Peptidase-IV Inhibitors of Natural Origin (Part I): Virtual Screening and Activity Assays
Source: PLoS One. 2012 Sep 12;7(9):e44971. doi: 10.1371/journal.pone.0044971 (PMC3440348; doi:10.1371/journal.pone.0044971)
Supplement: Table S1 — Predicted scaffold-hopping candidates for DPP-IV inhibition. This table shows ZINC codes for the 219 hit molecules predicted to inhibit DPP-IV that belong exclusively to clusters containing NPs that were previously unidentified as DPP-IV inhibitors. The best results of the shape and electrostatic-potential comparisons for each hit molecule with the ligand of 3C45 crystallized structure are shown. The Tanimoto values for the comparison between the electrostatic potentials of the molecules (using an outer dielectric of 80) are shown in the ET_PB columns. Furthermore, the values for the comparison between shapes are shown in the ET_Shape columns. The sum of the ET_PB and ET_Shape values is reported in the Combo columns. Hits from each cluster are sorted according to their decreasing combo value. ZINC00171758 and ZINC01833887 (cluster 30), ZINC02091906 (cluster 36), ZINC02118100 (cluster 37), ZINC02132035 and ZINC02160434 (cluster 41), ZINC02113150 (from cluster 45), ZINC00518468 (cluster 49) and ZINC02131242 (cluster 50) were tested in an in vitro assay to validate the success rate of our predictions (in bold in Table S1). Due to the insolubility, ZINC02118100 (cluster 37) and ZINC02160434 (cluster 41) could not be tested. (PDF) [file pone.0044971.s001.pdf]

Table S1

Compound	Cluster	ET_PB	ET_Combo	ET_Shape
ZINC08624216	10	0.670	1.299	0.629
ZINC08624227	10	0.664	1.270	0.606
ZINC12603399	10	0.660	1.120	0.460
ZINC08624212	10	0.679	1.063	0.384
ZINC12603352	10	0.647	1.039	0.393
ZINC12603382	10	0.628	1.009	0.381
ZINC08636132	10	0.737	1.007	0.270
ZINC04235298	29	0.732	1.113	0.381
ZINC03850486	29	0.629	1.011	0.383
ZINC01826519	30	0.691	1.390	0.699
ZINC01826518	30	0.716	1.254	0.538
ZINC02111476	30	0.747	1.252	0.505
ZINC02095165	30	0.702	1.237	0.535
ZINC02111654	30	0.680	1.221	0.541
ZINC04026917	30	0.766	1.174	0.408
ZINC04027780	30	0.745	1.167	0.422
ZINC00171740	30	0.764	1.166	0.402
ZINC02111107	30	0.689	1.159	0.470
ZINC01833886	30	0.703	1.157	0.454
**ZINC00171758**	**30**	**0.629**	**1.151**	**0.522**
ZINC02098064	30	0.736	1.136	0.400
ZINC01744114	30	0.733	1.135	0.402
ZINC01831741	30	0.666	1.133	0.467
ZINC00288906	30	0.661	1.130	0.469
ZINC01767546	30	0.688	1.123	0.435
**ZINC01833887**	**30**	**0.686**	**1.123**	**0.437**
ZINC01831742	30	0.660	1.121	0.461
ZINC02101423	30	0.687	1.120	0.433
ZINC04043691	30	0.720	1.119	0.398
ZINC01815758	30	0.626	1.117	0.490
ZINC00171737	30	0.770	1.113	0.344
ZINC00526001	30	0.669	1.113	0.444
ZINC04073289	30	0.699	1.109	0.411
ZINC02111026	30	0.626	1.102	0.476
ZINC00171675	30	0.681	1.101	0.420
ZINC04073208	30	0.662	1.091	0.428
ZINC00177015	30	0.760	1.089	0.330
ZINC00526000	30	0.646	1.089	0.443
ZINC02101849	30	0.697	1.089	0.392
ZINC01810429	30	0.690	1.079	0.389
ZINC04038362	30	0.721	1.075	0.353
ZINC01767542	30	0.731	1.073	0.342
ZINC02111059	30	0.644	1.065	0.421
ZINC02095167	30	0.655	1.049	0.394
ZINC01867331	30	0.669	1.047	0.378
ZINC01815759	30	0.682	1.044	0.362
ZINC00177014	30	0.672	1.041	0.369
ZINC00171760	30	0.654	1.040	0.386
ZINC01713519	30	0.655	1.033	0.378
ZINC01801287	30	0.631	1.023	0.392
ZINC02103306	30	0.636	1.019	0.384
ZINC00206548	30	0.664	1.005	0.341
ZINC01801292	30	0.629	0.993	0.364
ZINC04073422	30	0.635	0.939	0.304
ZINC08623233	30	0.626	0.890	0.263
ZINC02104434	36	0.779	1.260	0.481
ZINC02147315	36	0.792	1.206	0.414
ZINC02091908	36	0.711	1.202	0.490
**ZINC02091906**	**36**	**0.703**	**1.196**	**0.493**
ZINC02126019	36	0.808	1.196	0.389
ZINC02126020	36	0.791	1.163	0.372
ZINC04088482	36	0.692	1.117	0.425
ZINC02104432	36	0.741	1.108	0.367
ZINC02154701	36	0.684	1.093	0.409
ZINC02157136	36	0.722	1.060	0.337
ZINC04085398	36	0.682	1.059	0.377
ZINC04089120	36	0.644	1.042	0.398
ZINC02098258	36	0.691	1.041	0.350
ZINC02103154	36	0.624	1.019	0.394
ZINC04084395	36	0.694	1.017	0.323
ZINC08635889	36	0.688	1.007	0.319
ZINC04089122	36	0.678	1.005	0.327
ZINC04086470	36	0.655	0.928	0.273
ZINC02103096	37	0.783	1.290	0.507
ZINC02118102	37	0.808	1.230	0.421
ZINC02118103	37	0.770	1.213	0.443
**ZINC02118100**	**37**	**0.799**	**1.200**	**0.401**
ZINC02118098	37	0.766	1.192	0.427
ZINC00206863	37	0.713	1.181	0.468
ZINC00206867	37	0.757	1.167	0.410
ZINC00244210	37	0.700	1.166	0.466
ZINC00201903	37	0.718	1.150	0.431
ZINC01791910	37	0.641	1.139	0.498
ZINC04046504	37	0.777	1.123	0.346
ZINC00201907	37	0.736	1.121	0.385
ZINC00244221	37	0.770	1.117	0.347
ZINC02098134	37	0.683	1.117	0.433
ZINC04046709	37	0.745	1.114	0.369
ZINC00206865	37	0.768	1.106	0.337
ZINC04045984	37	0.644	1.092	0.448
ZINC02111169	37	0.735	1.082	0.347
ZINC04084607	37	0.673	1.070	0.397
ZINC02098138	37	0.648	1.054	0.406
ZINC04046507	37	0.787	1.052	0.264
ZINC00244212	37	0.716	1.051	0.335
ZINC00374801	37	0.732	1.034	0.301
ZINC02108985	37	0.673	1.015	0.342
ZINC04045983	37	0.743	1.013	0.270
ZINC04085193	37	0.633	1.009	0.375
ZINC00244216	37	0.678	1.006	0.328
ZINC04090129	37	0.658	0.964	0.306
ZINC00526580	38	0.724	0.968	0.244
ZINC03852206	38	0.632	0.967	0.335
ZINC05396688	40	0.663	1.261	0.598
ZINC08295759	40	0.669	1.160	0.491
ZINC05439072	40	0.715	1.097	0.382
ZINC05396078	40	0.624	1.096	0.472
ZINC03841441	40	0.704	1.073	0.369
ZINC03842017	40	0.682	1.073	0.392
ZINC05397647	40	0.707	1.069	0.362
ZINC05433763	40	0.660	1.055	0.395
ZINC03841985	40	0.690	1.034	0.344
ZINC05399378	40	0.624	1.033	0.410
ZINC05433915	40	0.675	1.032	0.357
ZINC05398720	40	0.643	1.029	0.386
ZINC05409706	40	0.700	1.001	0.301
ZINC05399865	40	0.663	0.974	0.312
ZINC05410357	40	0.630	0.971	0.341
ZINC04270597	40	0.634	0.970	0.336
ZINC05440675	40	0.657	0.928	0.271
ZINC05433913	40	0.631	0.898	0.267

Compound	Cluster	ET_PB	ET_Combo	ET_Shape
ZINC02138459	41	0.737	1.223	0.485
ZINC02117429	41	0.755	1.210	0.455
ZINC00407890	41	0.692	1.186	0.494
ZINC02122938	41	0.659	1.183	0.524
ZINC02114557	41	0.723	1.176	0.453
ZINC00132662	41	0.701	1.155	0.454
ZINC02125016	41	0.703	1.135	0.432
ZINC02124939	41	0.649	1.132	0.483
ZINC02159206	41	0.705	1.122	0.417
ZINC02125175	41	0.642	1.109	0.467
ZINC02159373	41	0.724	1.107	0.383
ZINC00934318	41	0.655	1.103	0.448
ZINC02124654	41	0.710	1.103	0.392
ZINC02159335	41	0.682	1.096	0.414
ZINC02122586	41	0.681	1.089	0.407
ZINC02123323	41	0.683	1.089	0.406
ZINC02135809	41	0.713	1.084	0.371
ZINC02123238	41	0.642	1.074	0.432
ZINC02124894	41	0.624	1.068	0.443
ZINC02123503	41	0.651	1.066	0.415
ZINC02112810	41	0.662	1.061	0.399
ZINC02115714	41	0.676	1.049	0.373
ZINC02120102	41	0.634	1.046	0.412
ZINC02117282	41	0.629	1.043	0.414
ZINC02112838	41	0.644	1.027	0.383
**ZINC02132035**	**41**	**0.690**	**0.983**	**0.293**
ZINC00037965	41	0.639	0.973	0.333
ZINC02119782	41	0.627	0.972	0.345
ZINC02131421	41	0.640	0.963	0.324
**ZINC02160434**	**41**	**0.658**	**0.963**	**0.306**
ZINC08298071	44	0.677	1.151	0.474
ZINC08300451	44	0.641	1.116	0.475
ZINC00920376	44	0.670	1.097	0.427
ZINC08623374	44	0.690	1.075	0.385
ZINC00978757	44	0.668	1.070	0.402
ZINC12602191	44	0.645	1.051	0.406
ZINC08298054	44	0.632	1.037	0.406
ZINC08300458	44	0.638	1.021	0.383
ZINC08254146	44	0.632	0.959	0.328
ZINC08254520	44	0.635	0.918	0.282
ZINC08300478	44	0.647	0.918	0.271
ZINC00940005	45	0.728	1.238	0.510
ZINC03847574	45	0.660	1.184	0.524
ZINC00703857	45	0.667	1.115	0.447
ZINC01628259	45	0.641	1.098	0.457
ZINC03846504	45	0.630	1.059	0.429
ZINC01679777	45	0.741	1.039	0.298
ZINC03847575	45	0.683	1.028	0.345
ZINC02122310	45	0.663	1.012	0.349
**ZINC02113150**	**45**	**0.626**	**0.965**	**0.339**
ZINC03846506	45	0.641	0.942	0.301
ZINC03846608	45	0.649	0.936	0.286
ZINC04267198	45	0.625	0.904	0.279
ZINC00518521	49	0.753	1.150	0.397
ZINC01824117	49	0.729	1.088	0.359
ZINC00518288	49	0.786	1.071	0.285
**ZINC00518468**	**49**	**0.726**	**1.070**	**0.344**
ZINC00518287	49	0.737	1.023	0.286
ZINC01760845	49	0.662	1.003	0.342
ZINC00518513	49	0.702	0.993	0.291
ZINC01790050	49	0.673	0.963	0.290
ZINC03736221	50	0.646	1.245	0.599
ZINC00526756	50	0.747	1.240	0.493
ZINC00281070	50	0.681	1.231	0.550
ZINC02138329	50	0.640	1.231	0.591
ZINC01322395	50	0.684	1.229	0.546
ZINC02131423	50	0.673	1.214	0.541
ZINC00526446	50	0.678	1.213	0.535
ZINC02128903	50	0.628	1.176	0.549
ZINC03736220	50	0.721	1.173	0.452
ZINC02114987	50	0.704	1.141	0.436
ZINC00386494	50	0.677	1.133	0.456
ZINC00131414	50	0.803	1.129	0.326
ZINC00188073	50	0.653	1.128	0.475
ZINC02100978	50	0.661	1.127	0.466
ZINC00189593	50	0.680	1.126	0.446
ZINC04028994	50	0.637	1.123	0.486
ZINC01322729	50	0.637	1.119	0.482
ZINC00281064	50	0.635	1.103	0.468
ZINC00276052	50	0.649	1.099	0.450
ZINC05954633	50	0.663	1.098	0.436
ZINC01824688	50	0.626	1.095	0.470
ZINC00386493	50	0.670	1.090	0.420
ZINC00386492	50	0.634	1.089	0.454
ZINC03736224	50	0.637	1.083	0.446
ZINC00756618	50	0.674	1.078	0.403
ZINC00060358	50	0.669	1.073	0.404
ZINC01825160	50	0.672	1.046	0.374
ZINC00978630	50	0.649	1.021	0.372
ZINC02135841	50	0.675	1.012	0.337
ZINC02096126	50	0.654	0.996	0.342
ZINC02128246	50	0.671	0.995	0.324
ZINC01838604	50	0.659	0.992	0.333
**ZINC02131242**	**50**	**0.644**	**0.990**	**0.346**
ZINC06624000	50	0.648	0.989	0.341
ZINC02102133	50	0.631	0.976	0.345
ZINC02131243	50	0.638	0.944	0.306
ZINC06624017	50	0.655	0.925	0.270
