# Supplementary material for: Identification of Novel Human Dipeptidyl Peptidase-IV Inhibitors of Natural Origin (Part I): Virtual Screening and Activity Assays
Source: PLoS One. 2012 Sep 12;7(9):e44971. doi: 10.1371/journal.pone.0044971 (PMC3440348; doi:10.1371/journal.pone.0044971)
Supplement: Table S2 — Docked fragments that have significant contributions to the GScore for XP descriptors that are 0.00 Kcal/mol for C5. The most potent DPP-IV inhibitor found by our dose-response studies (i.e., C5) has no contribution to the GScore by the following XP descriptors: (a) PhobEn (i.e., hydrophobic enclosure reward); (b) PhobEnHB (i.e., reward for hydrophobically packed H-bond); (c) PhobEnPairHB (i.e., reward for hydrophobically packed correlated H-bond); and (d) πCat (i.e., reward for π-cation interactions). This table displays the docked fragments showing the highest values for these XP descriptors and the common pharmacophore sites of Figure 3 that are matched to the corresponding fragment, if any. (DOC) [file pone.0044971.s002.doc]

**Table S2**. Docked fragments that have significant contributions to the GScore for XP descriptors that are 0.00 Kcal/mol for **C5**.

| **Fragment** | **Common pharmacophore site matched** | **PhobEn** | **PhobEnHB** | **PhobEnPairHB** | **πCat** |
| --- | --- | --- | --- | --- | --- |
| 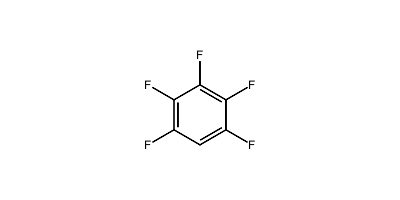 | H/R1 | -2.48 | 0.00 | 0.00 | 0.00 |
| 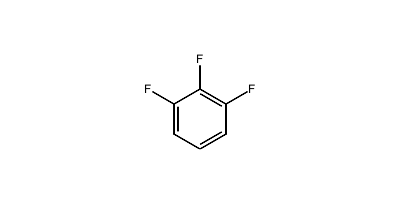 | H/R1 | -2.45 | 0.00 | 0.00 | 0.00 |
| 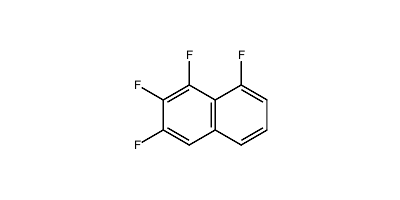 | H/R1 | -2.18 | 0.00 | 0.00 | 0.00 |
| 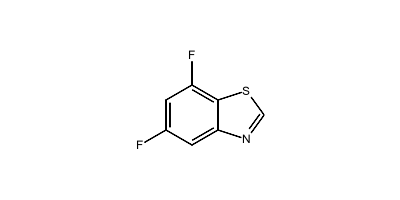 | H/R1 | -2.08 | 0.00 | 0.00 | 0.00 |
| 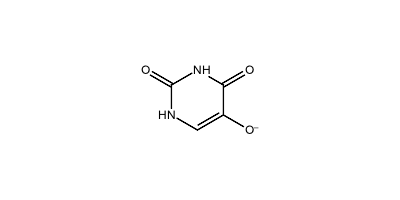 | H/R4 | 0.00 | -1.50 | 0.00 | 0.00 |
| 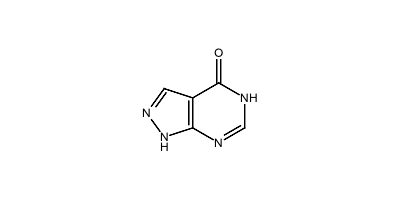 | H/R4 | 0.00 | -1.50 | 0.00 | 0.00 |
| 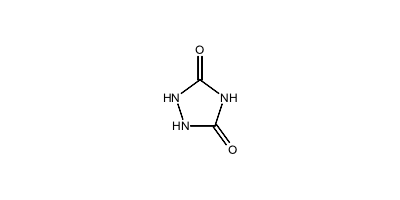 | H/R4 | 0.00 | -0.83 | 0.00 | 0.00 |
| 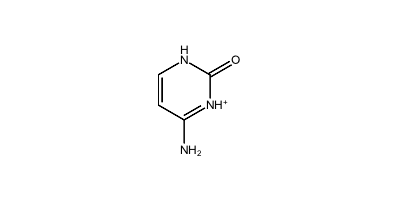 | H/R2 | 0.00 | 0.00 | -1.30 | 0.00 |
| 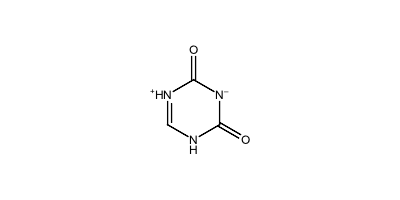 | H/R2 | 0.00 | 0.00 | -1.30 | 0.00 |
| 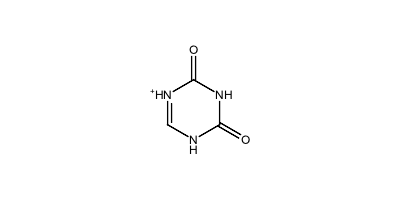 | H/R4 | 0.00 | 0.00 | -1.30 | 0.00 |
| 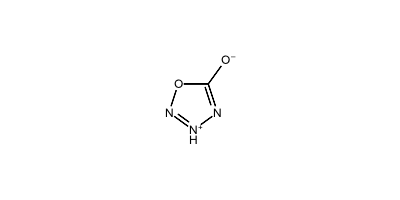 | H/R1 | -0.90 | 0.00 | 0.00 | -1.46 |
| 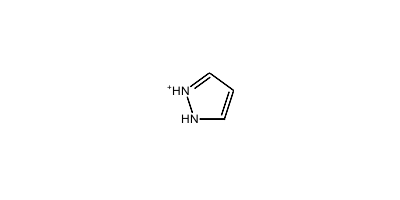 | H/R1 | 0.00 | 0.00 | 0.00 | -1.22 |
| 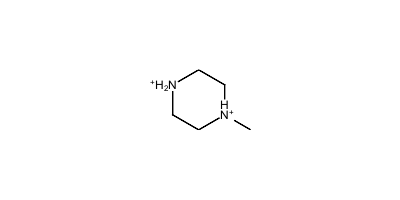 | (1) | 0.00 | 0.00 | 0.00 | -1.17 |

The most potent DPP-IV inhibitor found by our dose-response studies (*i.e.*, **C5**) has no contribution to the GScore by the following XP descriptors: **(a)** PhobEn (*i.e.*, hydrophobic enclosure reward); **(b)** PhobEnHB (*i.e.*, reward for hydrophobically packed H-bond); **(c)** PhobEnPairHB (*i.e.*, reward for hydrophobically packed correlated H-bond); and **(d)** πCat (*i.e.*, reward for π-cation interactions). This table displays the docked fragments showing the highest values for these XP descriptors and the common pharmacophore sites of Figure 3 that are matched to the corresponding fragment, if any.

(1) Although no pharmacophore site is matched to this fragment, it is located close to the **H/R1** site.
