# Supplementary material for: Identification of Novel Human Dipeptidyl Peptidase-IV Inhibitors of Natural Origin (Part I): Virtual Screening and Activity Assays
Source: PLoS One. 2012 Sep 12;7(9):e44971. doi: 10.1371/journal.pone.0044971 (PMC3440348; doi:10.1371/journal.pone.0044971)
Supplement: Table S3 — Top five C5 derivatives according to their XP GScores. The top five C5 derivatives according to their XP GScore values. The structures of the substituents that were attached to the C5 core at the two replacement sites (see Figure 5) are shown. The code for each molecule is obtained by adding the CombiGlide Diverse Side-chain Collection code for the substituents at the H/R1 and at the H/R2 sites to C5. (DOC) [file pone.0044971.s003.doc]

**Table S3**. Top five **C5** derivatives according to their XP GScores.

| **Code for C5 derivative** | **Substituents** | | **XP GScore (**Kcal/mol**)** |
| --- | --- | --- | --- |
|  | **H/R1** site | **H/R2** site |  |
| **C5-97-786** | 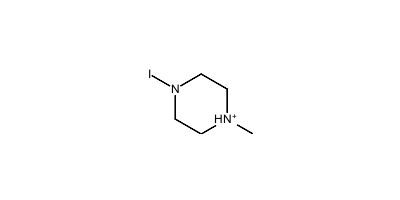 | 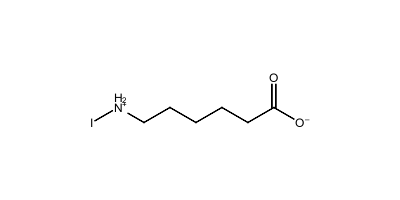 | -11.837 |
| **C5-137-784** | 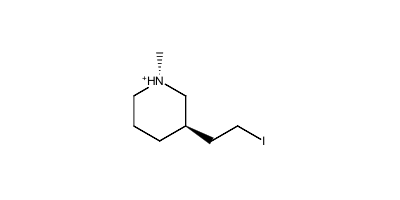 | 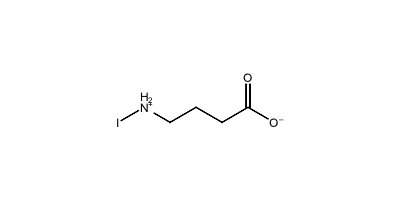 | -11.738 |
| **C5-100-563** | 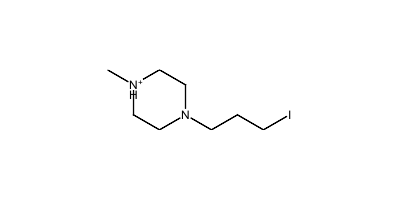 | 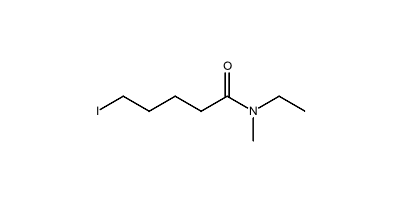 | -9.588 |
| **C5-309-787** | 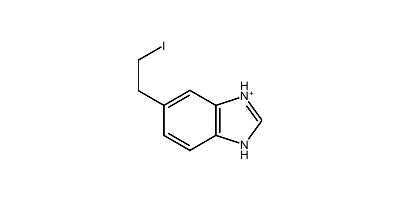 | 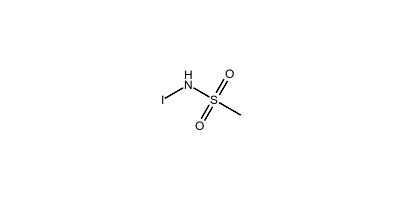 | -9.547 |
| **C5-274-536** | 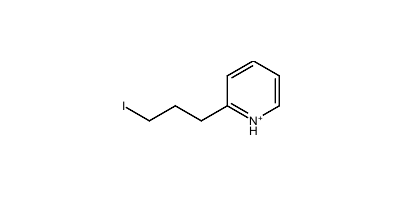 | 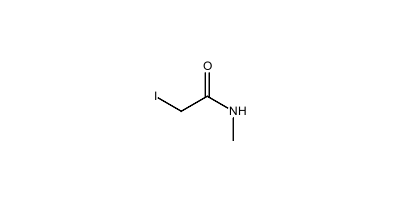 | -9.509 |

The top five **C5** derivatives according to their **XP GScore** values. The structures of the substituents that were attached to the **C5** core at the two replacement sites (see Figure 5) are shown. The code for each molecule is obtained by adding the CombiGlide Diverse Side-chain Collection code for the substituents at the H/R1 and at the H/R2 sites to **C5**.
